# Atomistic Modeling of Natural Gas Desulfurization Process Using Task-Specific Deep Eutectic Solvents Supported by Graphene Oxide

**DOI:** 10.3390/molecules29225282

**Published:** 2024-11-08

**Authors:** Olzhas Ismagambetov, Nakhypbek Aldiyarov, Nurlan Almas, Irina Irgibaeva, Zhadyra Baitassova, Sergei Piskunov, Anuar Aldongarov, Omirzak Abdirashev

**Affiliations:** 1 Department of Automation and Control, Satbayev University, Almaty 050000, Kazakhstan; olzhas.ismagambetov@gmail.com (O.I.); n.aldiyarov@satbayev.university (N.A.); 2 Department of Technical Physics, L. N. Gumilyov Eurasian National University, Astana 010000, Kazakhstan; nurlanalmasov@gmail.com (N.A.); irgsm@mail.ru (I.I.); baitasova_8855@mail.ru (Z.B.); 3 Institute of Solid State Physics, University of Latvia, 1000 Riga, Latvia; piskunov@lu.lv

**Keywords:** natural gas, desulfurization, task-specific DESs, graphene oxide, digital twin, density functional theory, molecular dynamics

## Abstract

This study employs Density Functional Theory (DFT) calculations and traditional all-atom Molecular Dynamics (MD) simulations to reveal atomistic insights into a task-specific Deep Eutectic Solvent (DES) supported by graphene oxide with the aim of mimicking its application in the natural gas desulfurization process. The DES, composed of N,N,N′,N′-tetramthyl-1,6-hexane diamine acetate (TMHDAAc) and methyldiethanolamine (MDEA) supported by graphene oxide, demonstrates improved efficiency in removing hydrogen sulfide from methane. Optimized structure and HOMO-LUMO orbital analyses reveal the distinct spatial arrangements and interactions between hydrogen sulfide, methane, and DES components, highlighting the efficacy of the DES in facilitating the separation of hydrogen sulfide from methane through DFT calculations. The radial distribution function (RDF) and interaction energies, as determined by traditional all-atom MD simulations, provide insights into the specificity and strength of the interactions between the DES components supported by graphene oxide and hydrogen sulfide. Importantly, the stability of the DES structure supported by graphene oxide is maintained after mixing with the fuel, ensuring its robustness and suitability for prolonged desulfurization processes, as evidenced by traditional all-atom MD simulation results. These findings offer crucial insights into the molecular-level mechanisms underlying the desulfurization of natural gas, guiding the design and optimization of task-specific DESs supported by graphene oxide for sustainable and efficient natural gas purification.

## 1. Introduction

Natural gas primarily consists of methane, a hydrocarbon with the chemical formula CH_4_. It may also contain other hydrocarbons and impurities, such as hydrogen sulfide. The presence of impurities in natural gas is crucial to monitor as they can affect its combustion properties, contribute to corrosion in pipelines and equipment, and pose safety risks. Controlling and removing impurities is essential for ensuring the efficient and safe use of natural gas in various applications, from residential heating to industrial processes. Hydrogen sulfide is a colorless gas with a flavor somewhat like sweet rotting eggs. Hydrogen sulfide is a strongly poisonous toxin that affects the nervous systems of humans. Concentrations as low as 0.2–0.3 mg/m^3^ can cause acute poisoning, and it can be lethal at concentrations higher than 1 mg/m^3^ [1,2,3,4,5,6]. In addition to being toxic, hydrogen sulfide functions as an acid and, in the presence of water, causes chemical and electrochemical corrosion. Furthermore, it can cause sulfide cracking in metals under certain circumstances, which puts materials exposed to this corrosive chemical at further danger.

In this regard, the extraction of hydrogen sulfide from natural gas is required by industrial requirements. These include (1) preventing corrosion in pipes, equipment, and facilities during gas transportation, processing, and utilization; (2) guaranteeing that the gas is appropriate for residential, commercial, and industrial uses; and (3) obtaining condensation products free of sulfur compound impurities by processing purified gasses. International regulations state that purified hydrocarbon gas may contain up to 0.02 g/m^3^ of hydrogen sulfide [6,7,8]. At this point, there are many methods for cleaning up hydrocarbon gas, which are generally divided into three categories: catalysis, adsorption, and absorption. The most common methods are those in the first group, which allows gas with different starting amounts of impurities to be treated. Adsorption techniques, on the other hand, are used to cope with lower initial impurity concentrations (about 3–5% vol.), and it has the benefit of providing complete gas purification.

Depending on the type of adsorbent used, adsorption techniques can be classified as chemical (chemisorption), physical, mixed, or oxidative. Amines are frequently used in chemisorption techniques, which entail the chemical interaction of hydrogen sulfide with the absorbent’s active ingredient. This procedure, which uses water as the chemisorbent and alkanolamine as the chemisorbent, is one famous example. Consequently, hydrocarbon gasses can be purified using chemisorption procedures, which remove acidic components such as hydrogen sulfide. Particular attention is placed on amine processes. Every approach has its own set of benefits and acknowledged disadvantages. In industrial contexts, the commercial and technical availability of amine has a major impact on the technique selection process. Furthermore, the absorption solution’s physicochemical characteristics are quite important when making decisions. When using the monoethanolamin technique, the chemisorbent is an aqueous solution of monoethanolamine that contains 15–30% by weight; larger concentrations of monoethanolamine are saved for circumstances in which the corrosion inhibitors are extremely efficient [7,8,9]. Monoethanolamine has some significant disadvantages, such as corrosive solutions and high reactivity towards organic sulfur compounds in gas. Moreover, in the presence of trace amounts of oxygen, monoethanolamine has been found to react with hydrogen sulfide to generate the non-regenerable molecule ethanolamine thiosulfate. In this regard, there are other new solvents that could be used for the desulfurization of natural gas, including ionic liquids and Deep Eutectic Solvents (DESs).

Ionic liquids are distinguished as sophisticated customizable solvents by virtue of their unique characteristics, which include a wide range of liquid temperatures, low volatility, and excellent thermal stability. However, their high cost, high viscosity, and complex preparation techniques pose hurdles to their widespread industrial application. Due to their many advantages—such as affordability, ease of preparation, very low volatility, and superior biocompatibility—Deep Eutectic Solvents (DESs) are thought to be a more environmentally friendly option [10,11,12,13,14]. Hydrogen bond interactions are usually used to combine hydrogen bond donors and hydrogen bond acceptors to generate DESs. Crucially, the kind and ratio of hydrogen bond components can be changed to customize their architectures, enabling the creation of unique DESs that are suited to a variety of industrial needs. Pudi et al. conducted a thorough review of technologies for capturing and removing hydrogen sulfide from natural gas, exploring diverse methods such as ionic liquids and DESs [4]. According to a review, regarding DES, task-specific DESs have a better hydrogen sulfide removal efficiency from natural gas. Namely, [C1-TMHDA]Ac-MDEA (1:2) surpassed all previously reported absorbents with an unparalleled hydrogen sulfide absorption capacity of 1.44 mol/mol at 313.2 K and 1.0 bar [15,16,17,18,19]. In addition, Xu and Hung reported that ethaline-based DESs confined by graphene oxide showed improved carbon dioxide removal from natural gas components [20]. Thorough knowledge of the absorption chemistry and natural gas sweetening processes is essential to carefully select components for task-specific DESs supported by graphene oxide with enhanced hydrogen sulfide.

In this work, the molecular interactions inside the task-specific DESs were examined, as well as the intermolecular interactions between task-specific DESs, hydrogen sulfide, and methane in the absence and presence of graphene oxide using Density Functional Theory (DFT) calculations and traditional all-atom Molecular Dynamics (MD) simulations. In particular, using known experiments and modeling settings, [C1-TMHDA]Ac-MDEA (1:1) was selected to study the natural gas upgrading process. In addition, this study explores how newly designed task-specific DES supported by graphene oxide affect the natural gas sweetening process via DFT calculations and traditional all-atom MD simulations. Based on absorption mechanisms and simulation insights, this study offers well-informed guidance for creating unique task-specific DESs supported by graphene oxide for natural gas desulfurization.

## 2. Results and Discussion

### 2.1. DFT Results

#### 2.1.1. Formation of Task-Specific DES at the DFT Level

Task-specific DESs have emerged as versatile and sustainable alternatives in various applications owing to their unique properties. This study employs DFT calculations to investigate the intermolecular formation of a task-specific DES. The focus lies on elucidating the optimized structures of key components and the resultant DES, as well as exploring the energetics and electronic properties through HOMO and LUMO orbitals.

**Figure 1:**
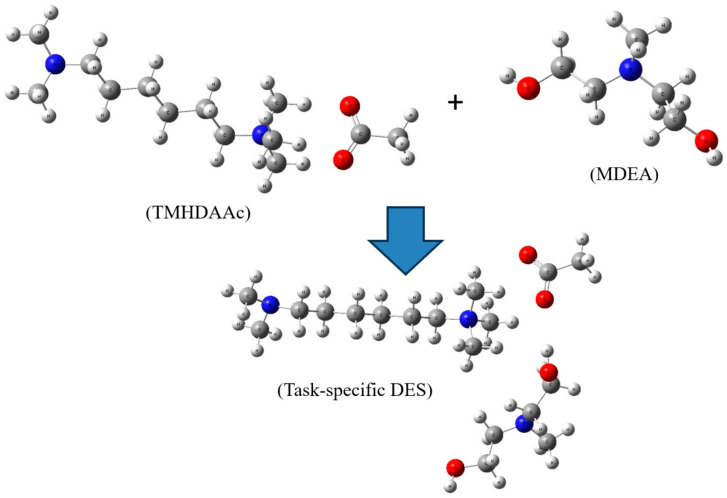
Optimized structures of TMHDAAc, MDEA, and task-specific DES. Color legend: white represents hydrogen; gray indicates carbon; blue denotes nitrogen; and red signifies oxygen.

Figure 1 presents a crucial snapshot of the molecular landscape, showcasing the optimized structures of TMHDAAc, MDEA, and the task-specific DES. In the optimized TMHDAAc, intricate interactions unfold between TMHDA and Ac, with the tetramethyl group of TMHDA engaging with the acetyl group of Ac. This initial configuration sets the stage for further chemical transformations. Upon introducing MDEA into the system, novel chemical interactions come into play. Figure 1 illustrates the evolution of the molecular architecture, where the TMHDA tetramethyl group and acetyl group of acetate not only maintain their interactions but also form new bonds with the hydroxyl group of MDEA. This dynamic interplay signifies the formation of a complex structure, suggesting the potential development of enhanced properties in the resulting DES.

A crucial aspect of understanding the intermolecular interactions in DES lies in the energetic considerations. DFT optimized energy calculations provide insights into the stability and favorability of molecular configurations. The energy landscape of the task-specific DES and its individual components is meticulously examined, shedding light on the thermodynamic feasibility of the intermolecular formations observed in Table 1.

Table 1 provides a succinct overview of the optimized energy values for the key components involved in the formation of the task-specific DES. The energy for TMHDAAc is optimized at −21,050.72 eV, representing the energy associated with the stable molecular configuration of this component. Likewise, MDEA shows an optimized energy of −10,983.88 eV, offering an insight into the stability of its structure. These individual energy values serve as reference points, laying the foundation for understanding their contributions to the overall energy landscape of the system.

The task-specific DES, resulting from the synergistic interactions between TMHDAAc and MDEA, has an optimized energy of −32,034.43 eV. This composite energy value reflects the total energy contributions from intermolecular forces and chemical bonding within the DES. The negative energy value underscores the system’s stability, suggesting that the formation of the task-specific DES is energetically favorable.

The excess energy is minimal at 0.17 eV, further indicating that the stability of the DES is primarily governed by the major energy contributions from the individual components. This small amount of excess energy highlights the stability of the DES as it is primarily governed by the substantial energy contributions from TMHDAAc and MDEA. The revised table presents these values with enhanced clarity for better readability and analysis.

Additionally, the bond lengths between significant interacting atoms are included: the bond length between the oxygen atom of Ac and the hydrogen atom of TMHDA is measured at 2.17 Å, while the bond length between the oxygen atom of MDEA and the hydrogen atom of TMHDA is 2.62 Å (Table 1). Furthermore, the bond length between the oxygen atom of Ac and the hydrogen atom of MDEA is 2.44 Å. These bond lengths provide valuable insights into the interactions within the DES system, allowing for a more detailed understanding of the structural characteristics that contribute to its stability.

**Figure 2:**
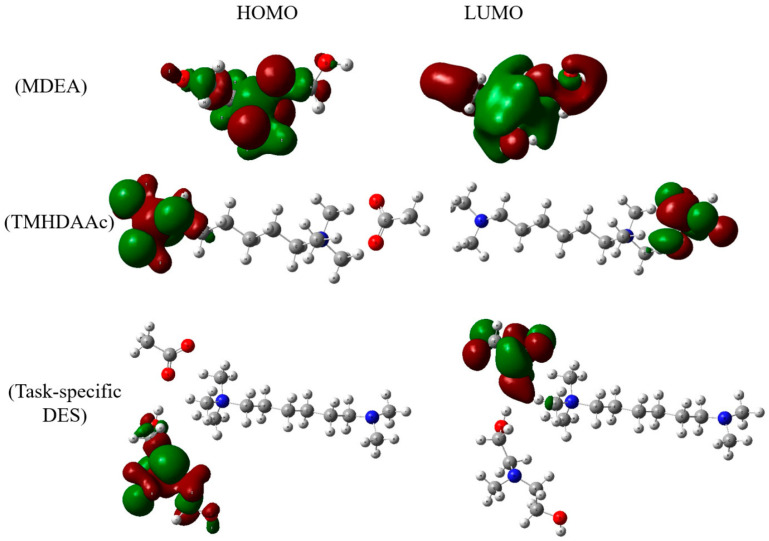
HOMO-LUMO orbitals of MDEA, TMHDAAc, and task-specific DES. Color legend: white represents hydrogen; gray indicates carbon; blue denotes nitrogen; and red signifies oxygen. Dark green and dark red indicate the locations of molecular orbitals.

The electronic structure of the DES and its components is explored through HOMO and LUMO orbitals. These orbitals play a pivotal role in dictating the reactivity and electronic properties of the system. Figure 2 captures the spatial distributions of these orbitals, offering a comprehensive understanding of the electronic characteristics associated with the intermolecular interactions.

Figure 2 provides a visual representation of the HOMO and LUMO orbitals for MDEA, TMHDAAc, and the task-specific DES. The energy levels associated with these orbitals are crucial in understanding the electronic characteristics and reactivity of the molecules.

For TMHDAAc, the HOMO is recorded at −8.33 eV, indicating the energy level of the highest occupied orbital, while the LUMO is observed at −2.70 eV, representing the lowest unoccupied orbital. This energy gap provides insights into the electron donation and acceptance capabilities of TMHDAAc, influencing its chemical reactivity. In the case of MDEA, the HOMO is positioned at −8.23 eV, and the LUMO is found at −1.39 eV. These values contribute to understanding the electron density distribution and potential electron acceptance or donation by MDEA, guiding its interactions in chemical processes. When examining the task-specific DES, the HOMO is situated at −8.20 eV, and the LUMO is identified at −2.81 eV. These orbital energies reveal the electronic characteristics specific to the DES, providing insights into its reactivity and stability. The comparison of the HOMO and LUMO energies among the individual components aids in understanding the changes in the electronic structure upon the formation of the task-specific DES. Figure 2 and the associated HOMO-LUMO energy values present a comprehensive view of the electronic structure of MDEA, TMHDAAc, and the task-specific DES. These insights are essential for elucidating the reactivity and potential applications of the studied molecules in various chemical processes.

#### 2.1.2. The Desulfurization Process in the Presence of the Task-Specific DES at the DFT Level

**Figure 3:**
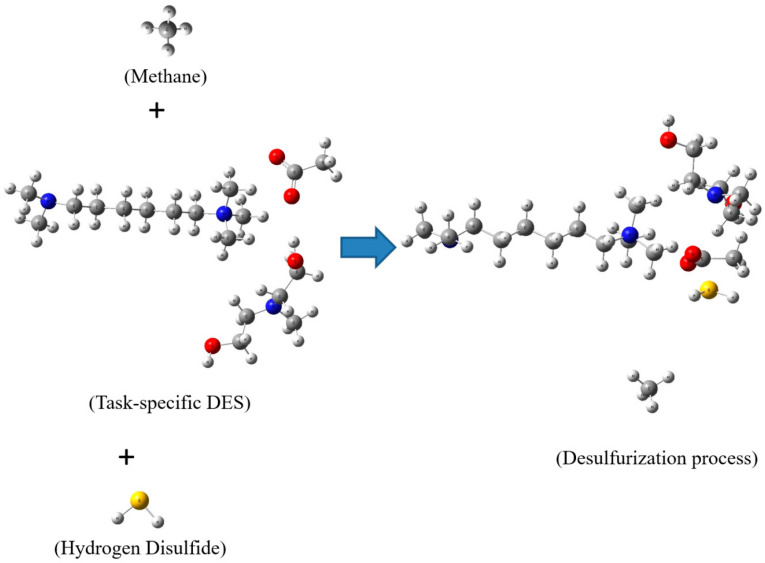
Optimized structures of task-specific DES, hydrogen sulfide, methane, and desulfurization process. Color legend: white represents hydrogen; gray indicates carbon; blue denotes nitrogen; yellow denotes sulfur; and red signifies oxygen.

This study employs DFT calculations to investigate the desulfurization process of natural gas in the presence of a task-specific DES. Figure 3 showcases optimized structures of methane, hydrogen sulfide, and the task-specific DES, providing a molecular-level understanding of the interactions involved in the desulfurization process.

Figure 3 presents a comprehensive view of the molecular landscape, revealing the optimized structures of key components—methane, hydrogen sulfide, and the task-specific DES. The focus then shifts to the chemical interactions occurring after mixing TMHDAAc with MDEA. The newly formed interactions between the TMHDA tetramethyl group, the acetyl group of acetate, and the MDEA hydroxyl group play a pivotal role in facilitating the desulfurization process.

One noteworthy finding illustrated in Figure 3 is the chemical absorption of hydrogen sulfide facilitated by the task-specific DES. The acetyl group of Ac within the DES exhibits a chemical affinity to absorb hydrogen sulfide, forming a stable complex with a distance of 3.32 Angstroms. This proximity indicates a favorable chemical interaction between the acetyl group and hydrogen sulfide, providing insights into the efficiency of the desulfurization process. In contrast to hydrogen sulfide, methane exhibits a different spatial arrangement within the DES. The optimized structure reveals that methane is located at a distance of 6.38 Angstroms from the DES. This spatial separation implies a weaker interaction between methane and the DES compared to hydrogen sulfide. Understanding the distinct spatial distributions of these components is crucial for assessing the selectivity and efficiency of the desulfurization process.

The acetyl group of Ac within the DES exhibits a chemical affinity to absorb hydrogen sulfide, forming a stable complex with a distance of 3.32 Å. This distance, being slightly greater than the typical distance observed in Coulombic repulsion (~3.0 Å), confirms that the interaction is intermolecular rather than intramolecular. The intermolecular nature of this interaction suggests that the acetyl group is not involved in internal bonding within the molecule but instead forms an external bond with hydrogen sulfide. This evidence underscores the efficiency of the DES in facilitating the adsorption of hydrogen sulfide through favorable interactions. Additional support for this intermolecular interaction is provided by the data presented in later subsections, which collectively demonstrate the role of graphene oxide in enhancing these interactions. The analysis of these figures offers further evidence of the DES and graphene oxide system’s potential for the efficient desulfurization of natural gas through stable and efficient adsorption processes.

The observed chemical interactions and spatial arrangements shed light on the potential of the task-specific DES in the desulfurization of natural gas. The selective absorption of hydrogen sulfide by the acetyl group of Ac indicates a targeted approach, allowing for the removal of sulfur-containing impurities while maintaining a controlled interaction with other gas components such as methane. This selectivity is essential for the development of efficient and environmentally friendly desulfurization processes. The DFT calculations presented in this study provide valuable insights into the desulfurization process of natural gas using a task-specific Deep Eutectic Solvent. Figure 3 elucidates the optimized structures of methane, hydrogen sulfide, and the DES, highlighting the crucial chemical interactions that drive the desulfurization process. The specific affinity of the acetyl group of Ac within the DES for hydrogen sulfide, coupled with the spatial distribution of methane, indicates the potential for a selective and efficient desulfurization process. This molecular-level understanding contributes to the ongoing efforts in developing sustainable and effective methods for natural gas desulfurization.

**Figure 4:**
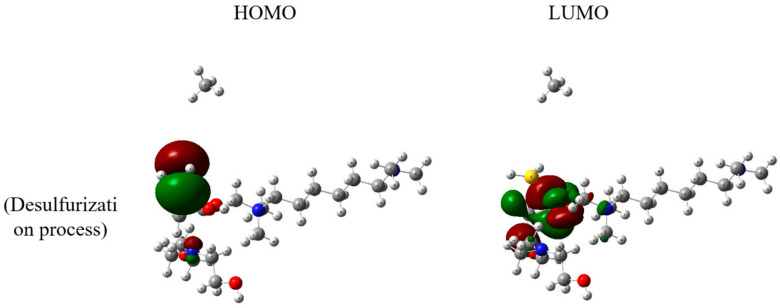
HOMO-LUMO orbitals of desulfurization process in presence of methane, hydrogen sulfide, and task-specific DES. Color legend: white represents hydrogen; gray indicates carbon; blue denotes nitrogen; yellow denotes sulfur; and red signifies oxygen. Dark green and dark red indicate the locations of molecular orbitals.

The HOMO and LUMO orbitals of the desulfurization process in the presence of methane, hydrogen sulfide, and the task-specific DES provide crucial insights into the electronic structure and reactivity of the system. Specifically, the HOMO-LUMO orbitals of the task-specific DES play a central role in understanding the interactions involved in the desulfurization process (Figure 4). For the task-specific DES, the HOMO orbital is calculated at −7.83 eV, reflecting the energy level of the highest occupied orbital. Simultaneously, the LUMO orbital is positioned at −2.84 eV, representing the lowest unoccupied orbital. These energy values are instrumental in understanding the electron distribution and availability for chemical interactions within the DES during the desulfurization process. The HOMO-LUMO orbitals of the task-specific DES offer insights into its electronic characteristics and reactivity. The energy gap between the HOMO and LUMO orbitals influences the DES’s capability to donate or accept electrons, contributing to its role in the chemical absorption of hydrogen sulfide and the overall desulfurization process.

These orbital energy values provide a foundation for comprehending the electronic aspects of the task-specific DES and its interactions with methane and hydrogen sulfide. The HOMO-LUMO analysis is essential for evaluating the electronic properties that drive the reactivity and selectivity of the DES in the context of natural gas desulfurization. Overall, the presented orbital data contribute to a deeper understanding of the electronic dynamics that underlie the efficiency and specificity of the desulfurization process facilitated by the task-specific Deep Eutectic Solvent.

### 2.2. Classical All-Atom MD Simulations

#### 2.2.1. Formation of Task-Specific DES at the MD Level

**Figure 5:**
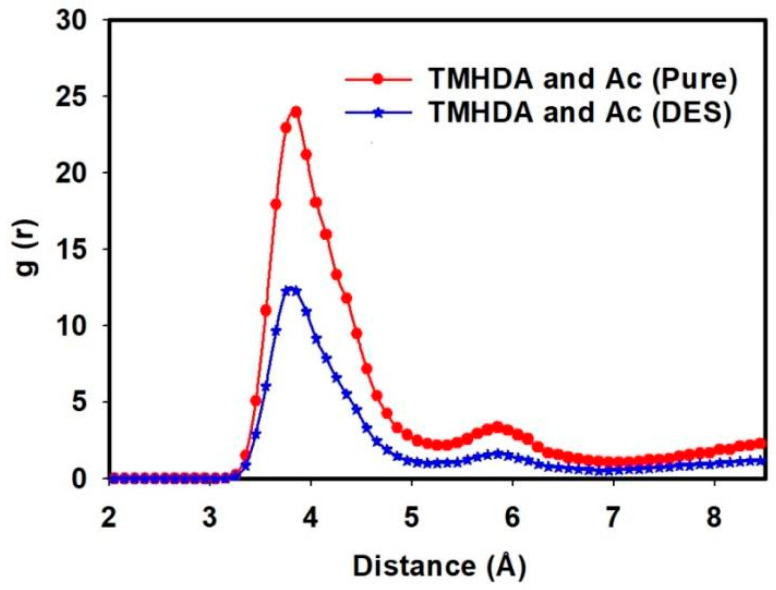
The RDF between TMHDA and Ac in the pure case and in the presence of MDEA as a DES component.

Figure 5 and the accompanying tables provide a detailed analysis of the intermolecular interactions within the task-specific DES system, shedding light on the impact of MDEA as a component. The RDF is employed to visualize the spatial distribution of TMHDA and Ac, while interaction energies and hydrogen bonding data offer quantitative insights into the molecular dynamics.

Figure 5 illustrates an initial intermolecular interaction between TMHDA and Ac at 23.95 Angstrom, with a peak occurrence at 3.85 Angstrom, in the pure TMHDAAc case. However, upon the introduction of MDEA and the formation of the DES, this interaction distance significantly decreases to 12.27 Angstrom at 3.85 Angstrom. This observed reduction suggests a closer association between TMHDA and Ac in the presence of MDEA, indicating the influence of MDEA on the intermolecular arrangement within the DES.

Table 2 provides quantitative data on the interaction energies between TMHDA and Ac in pure TMHDAAc and after mixing with MDEA. In the pure case, the interaction energy is recorded at −10,843.2 kJ/mol, while in the presence of MDEA, the energy decreases to −9895.71 kJ/mol. This reduction in interaction energy further supports the notion that the incorporation of MDEA alters the intermolecular forces within the DES, influencing the stability of the TMHDA-Ac interaction.

Table 3 delves into the number of hydrogen bonds between the DES and its components. In pure TMHDAAc, the interaction energy between TMHDA and Ac is characterized by 690 hydrogen bonds. After mixing with MDEA, this number decreases to 612. The decline in the number of hydrogen bonds suggests a modification in the bonding pattern within the DES, indicating the role of MDEA in reshaping the hydrogen bonding network.

The data in Table 3 highlight a noticeable difference in the numbers of hydrogen bonds between the DES components, particularly between TMHDA and MDEA. The interaction energy between TMHDA and Ac in pure TMHDAAc is supported by 690 hydrogen bonds, but this number decreases to 612 in the DES mixture. Notably, the number of hydrogen bonds between TMHDA and MDEA is significantly lower at 63. This lower frequency of hydrogen bonding between TMHDA and MDEA compared to other components may indicate that MDEA interacts differently within the DES. The reduced hydrogen bonding could suggest a partial dissociation or weaker interaction between MDEA and the TMHDAAc complex. This may lead MDEA to form fewer hydrogen bonds with TMHDA while actively participating in other bonding interactions within the system, as seen in the increased bonding between MDEA and Ac (383 hydrogen bonds). Such reshuffling of the hydrogen bonding network is characteristic of DES systems, where different components compete for bonding sites, altering the overall bonding dynamics.

The combined analysis of RDF, interaction energies, and hydrogen bonding data provides a comprehensive understanding of the intermolecular interactions within the task-specific DES. The decreases in interaction distances, energy values, and hydrogen bond counts upon the introduction of MDEA highlight its influence on the molecular architecture. These findings contribute to the broader comprehension of how the addition of specific components can fine-tune the interactions within a DES, potentially impacting its stability and performance in various applications. This study sets the stage for further investigations into the design and optimization of a task-specific DES for specific industrial processes.

This study delves into the intermolecular interactions within a task-specific DES, focusing on the RDF, interaction energies, and hydrogen bonding. By examining the relationships between TMHDA and Ac, MDEA and Ac, and TMHDA and MDEA, we gain comprehensive insights into the spatial arrangements, energetics, and bonding patterns that characterize the DES.

**Figure 6:**
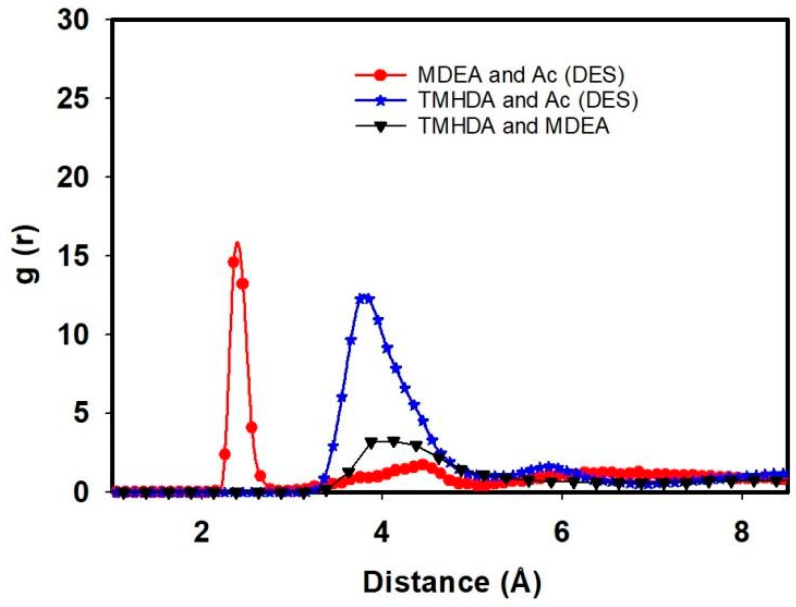
RDF between TMHDA and Ac, MDEA and Ac, and TMHDA and MDEA as DES component.

The RDF analysis reveals compelling insights into the spatial distribution of molecular pairs within the DES. After the mixing of TMHDAAc with MDEA, the RDF between TMHDA and Ac decreases to 12.27 at 3.85 Angstrom, signifying a closer association. Simultaneously, the RDF between MDEA and Ac is 14.58 at 2.35 Angstrom, indicating a strong interaction in the vicinity. The RDF between TMHDA and MDEA is 3.26 at 4.12 Angstrom, underscoring the distinct spatial arrangement between these two components within the DES. These observations highlight the dynamic nature of intermolecular interactions in the presence of MDEA (Figure 6).

Table 2 presents the interaction energies within the DES, providing a quantitative measure of the stability of the molecular configurations. The interaction energy between TMHDA and Ac within the DES is −9895.71 kJ/mol, reflecting a favorable interaction between these components. The TMHDA and MDEA interaction energy is −1828.51 kJ/mol, indicating a stabilizing force between these components. The MDEA and Ac interaction energy is −10,783.8 kJ/mol, suggesting a strong affinity between MDEA and Ac within the DES. These values emphasize the intricate balance of forces contributing to the overall stability of the DES.

Table 3 provides insights into the hydrogen bonding patterns within the DES, which are crucial for understanding the cohesive forces between molecules. The number of hydrogen bonds between TMHDA and Ac within the DES is 612, underscoring a complex network of interactions. The TMHDA and MDEA interaction is characterized by 63 hydrogen bonds, indicative of a more localized and specific bonding pattern. The MDEA and Ac interaction involves 383 hydrogen bonds, reflecting a rich network of interactions between MDEA and Ac within the DES. These hydrogen bonding patterns contribute significantly to the overall stability and functionality of the DES.

The integrated analysis of RDF, interaction energies, and hydrogen bonding patterns provides a comprehensive understanding of the intermolecular dynamics within the task-specific DES. The decrease in RDF distances, diverse interaction energies, and intricate hydrogen bonding networks underscore the complexity and versatility of the DES system. These findings pave the way for the further exploration and optimization of task-specific DESs for tailored applications, ranging from chemical processes to sustainable solvent systems.

#### 2.2.2. The Desulfurization Process in the Presence of the Task-Specific DES at the MD Level

**Figure 7:**
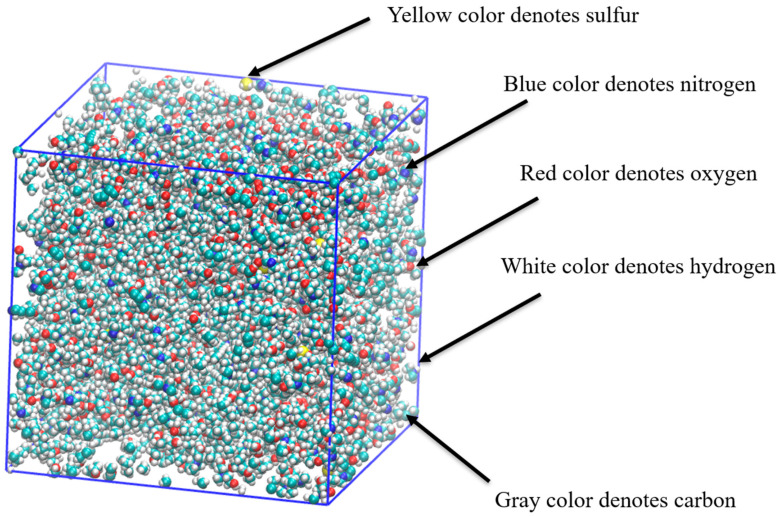
Snapshot of MD simulation of desulfurization process of natural gas using task-specific DES. Color legend: white represents hydrogen; gray indicates carbon; blue denotes nitrogen; yellow denotes sulfur; and red signifies oxygen.

In this subsection, we delve into the classical all-atom MD simulations focused on the desulfurization process of natural gas utilizing a task-specific DES. Specifically, the removal of hydrogen sulfide from methane is examined through the combination of TMHDAAc and MDEA in the task-specific DES. Figure 7 provides a snapshot of the MD simulation, offering a visual representation of the dynamic interactions during the desulfurization process.

**Figure 8:**
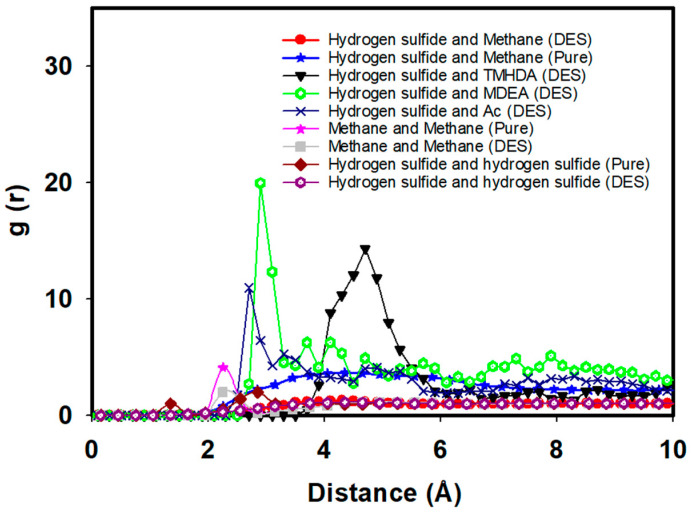
RDFs between hydrogen sulfide and DES components.

To gain deeper insights into the molecular interactions, Figure 8 illustrates the RDF between hydrogen sulfide and various components, including methane and DES constituents. In the pure fuel case before mixing with the task-specific DES, the RDF peak value between hydrogen sulfide and methane is observed at 3.67 with a distance of 4.35 Angstrom. This initial RDF peak signifies the natural association of hydrogen sulfide with methane in the absence of the DES.

Following the introduction of the task-specific DES to the fuel mixture (methane + hydrogen sulfide), significant changes in RDF patterns are observed. The RDF between hydrogen sulfide and methane decreases to 1.24 at 4.10 Angstrom, indicating a notable reduction in their association (Figure 8). This suggests an effective separation of hydrogen sulfide from methane facilitated by the task-specific DES. Moreover, the RDF between methane–methane interactions reveals a decrease after the addition of the task-specific DES, with the peak value reducing significantly. This indicates that the introduction of DES disrupts the natural association between methane molecules, enhancing the separation of methane from hydrogen sulfide. Similarly, for H_2_S-H_2_S interactions, the RDF shows a decreased intensity, suggesting reduced hydrogen sulfide clustering due to the DES’s influence, which promotes more effective dissociation within the system.

Furthermore, new interactions emerge between hydrogen sulfide and DES components after mixing. The RDF between hydrogen sulfide and Ac is noted at 10.97 at 2.7 Angstrom, indicating a close proximity and a strong interaction between hydrogen sulfide and the acetyl group. The RDF between hydrogen sulfide and TMHDA is observed at 14.31 at 4.7 Angstrom, highlighting a distinct spatial arrangement and interaction between these components. Additionally, the RDF between hydrogen sulfide and MDEA is recorded at 6.26 at 3.7 Angstrom, suggesting a specific binding pattern between hydrogen sulfide and the hydroxyl group of MDEA. These findings emphasize the role of the task-specific DES in mediating targeted interactions for efficient desulfurization.

**Figure 9:**
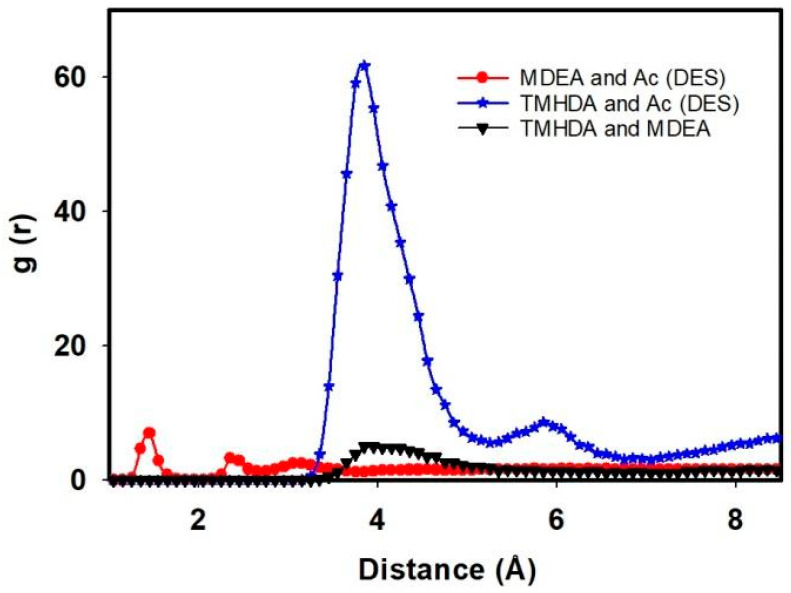
RDF between DES components during desulfurization of natural gas.

An essential aspect of the desulfurization process is the stability of the task-specific DES structure after mixing with the fuel. The RDF analysis between DES components reveals that the structure remains unchanged, indicating the robust stability of the DES. After mixing the DES with fuel, the RDF between DES components, namely, Ac and TMHDA, is recorded at 61.66 at 3.85 Angstrom, highlighting the stability of the interaction between the acetyl group and TMHDA. The RDF between Ac and MDEA is 6.96 at 1.55 Angstrom, indicating a stable interaction between the acetyl group and MDEA. The RDF between MDEA and TMHDA is 5.03 at 3.85 Angstrom, demonstrating a consistent arrangement between MDEA and TMHDA within the DES structure. These results underscore the robustness of the task-specific DES, ensuring its effectiveness and longevity in the desulfurization process (Figure 9).

The MD simulations and RDF analyses provide valuable insights into the desulfurization process of natural gas using a task-specific DES comprising TMHDAAc and MDEA. The reduction in RDF values between hydrogen sulfide and methane, coupled with the emergence of new interactions with DES components, showcases the efficiency of the DES in selectively removing hydrogen sulfide. Importantly, the stability of the task-specific DES structure post-mixing with the fuel emphasizes its resilience and suitability for sustained desulfurization processes. This molecular-level understanding contributes to the ongoing efforts in designing and optimizing task-specific DESs for practical applications in the clean energy sector.

Table 4 provides valuable insights into the interaction energies within the task-specific DES before and after its interaction with the fuel mixture. In the pure fuel case, the interaction energy between hydrogen sulfide and methane is recorded at −17.23 kJ/mol. Post-mixing with the DES, this energy is significantly reduced to −0.82 kJ/mol, highlighting the efficiency of the DES in facilitating the separation of hydrogen sulfide from methane.

Crucially, new interactions emerge between hydrogen sulfide and DES components after mixing with fuel. The interaction energy between hydrogen sulfide and Ac is −135.24 kJ/mol, emphasizing a strong affinity between these entities. Similarly, the interaction energy between hydrogen sulfide and TMHDA is −11.96 kJ/mol, while with MDEA, it is −38.21 kJ/mol. These interaction energy values underscore the specificity and effectiveness of the task-specific DES in mediating targeted interactions during the desulfurization process.

Table 4 also demonstrates that the DES structure remains unchanged after mixing with fuel, indicating the stability of the DES. The interaction energies between DES components, namely Ac and TMHDA (−10,403.53 kJ/mol), Ac and MDEA (−6858.04 kJ/mol), and MDEA and TMHDA (−1367.23 kJ/mol), remain consistent with the previous case. This stability reinforces the resilience of the DES structure, ensuring its effectiveness and suitability for sustained desulfurization processes. The combined analysis of interaction energies and structural stability in the presence of the fuel mixture validates the potential of the task-specific DES for practical applications in hydrogen sulfide removal from natural gas.

Table 5 provides crucial insights into the stability of the task-specific DES structure after its interaction with the fuel mixture. The number of hydrogen bonds between DES components, specifically Ac and TMHDA, Ac and MDEA, and MDEA and TMHDA, remains consistent after mixing with the fuel. The recorded values of 670, 260, and 590, respectively, mirror those observed in the previous case before fuel interaction. This consistent hydrogen bonding pattern signifies the robustness and stability of the DES structure, reinforcing its effectiveness in maintaining molecular integrity even in the presence of the fuel mixture. The preservation of hydrogen bonding interactions within the DES components underscores its resilience and suitability for prolonged desulfurization processes, further validating its potential for practical applications in the removal of hydrogen sulfide from natural gas. These DFT calculations, followed by all-atom MD simulations, could be further modeled with a digital twin for the desulfurization process of natural gas in the oil and gas industry and for water splitting processes in the future.

#### 2.2.3. The Desulfurization Process in the Presence of a Task-Specific DES Supported by Graphene Oxide at the MD Level

**Figure 10:**
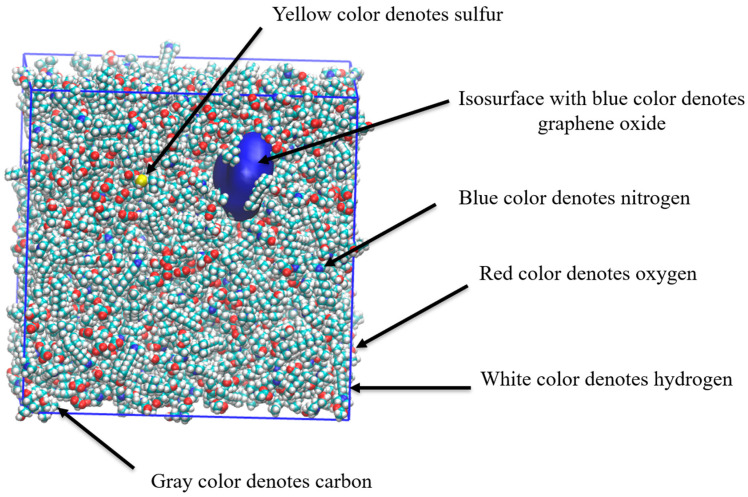
Snapshot of MD simulation of desulfurization process of natural gas by task-specific DES supported by graphene oxide. Color legend: white represents hydrogen; gray indicates carbon; blue denotes nitrogen; yellow denotes sulfur; isosurface with blue color denotes graphene oxide; and red signifies oxygen.

**Figure 11:**
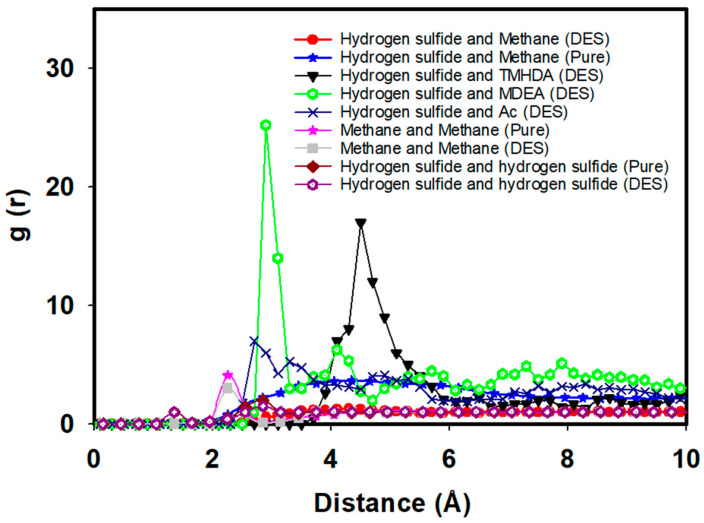
RDFs between hydrogen sulfide and DES components supported by graphene oxide.

**Figure 12:**
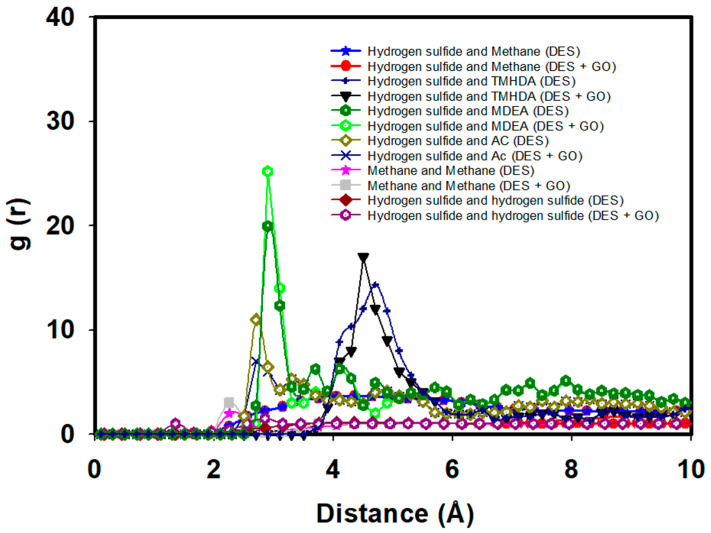
RDFs between hydrogen sulfide and DES components in absence and presence of graphene oxide (GO).

The present study investigates the desulfurization of natural gas using a task-specific DES system supported by graphene oxide with a focus on the interactions between hydrogen sulfide and DES components in the presence of methane. This discussion analyzes the key findings from the MD simulations, as evidenced by Figure 10, Figure 11 and Figure 12 and Table 6 and Table 7.

Figure 10 presents a visual snapshot of the MD simulation, which highlights the interactions during the desulfurization process. The image clearly shows that the graphene oxide, represented by the blue surface, is enveloped by the DES molecules. This encapsulation is a significant observation as it suggests that the DES molecules are effectively interacting with the graphene oxide’s surface, leading to a stable structure. Additionally, the DES molecules appear to cover both hydrogen sulfide and methane, indicating that the DES system is capable of interacting with these gas molecules, potentially facilitating the desulfurization process.

The RDFs provide critical insights into the molecular interactions between hydrogen sulfide and the DES components, as well as methane, in the presence of graphene oxide. As seen in Figure 12, the RDF between hydrogen sulfide and methane shows a lower peak value around 1, indicating weaker interactions in the presence of DES. In the presence of graphene oxide, as seen in Figure 12, both methane–methane and H_2_S-H_2_S interactions exhibit similar trends, with further reductions in their RDF peaks. This reflects weaker interactions, demonstrating the combined effect of graphene oxide and DES in decreasing the associations between methane and hydrogen sulfide, leading to even higher efficiency. This is further supported by the adsorption energy data in Table 6, where the interaction energy between hydrogen sulfide and methane in the presence of DES is significantly reduced to −0.56 kJ/mol compared to −17.23 kJ/mol in the pure system. This reduction in interaction strength suggests that the DES components effectively mitigate the affinity between hydrogen sulfide and methane, allowing for more selective desulfurization.

The RDF between hydrogen sulfide and MDEA shows a peak value of 25.21 at around 3.5 angstroms, indicating a strong interaction between these two molecules (Figure 12. This interaction is essential for the effective capture of hydrogen sulfide, as MDEA is known for its role in acid gas removal processes. Similarly, the RDF between hydrogen sulfide and acetate shows a peak value of 7 at 3.5 angstroms, suggesting that acetate also plays a significant role in interacting with hydrogen sulfide. The RDF between hydrogen sulfide and TMHDA (tetramethylhexanediamine) peaks at around 18 at 5 angstroms, indicating that this component also contributes to the overall interaction with hydrogen sulfide, although at a slightly greater distance than MDEA and acetate.

We overlaid the RDF curves from Figure 9 and Figure 12, as seen in Figure 12. This allowed us to highlight any potential effects of graphene oxide on the interactions between methane, hydrogen sulfide, and the DES components. The presence of graphene oxide slightly diminishes the peak intensity for methane–hydrogen sulfide interactions, indicating weaker interactions compared to the scenario without graphene oxide. These findings align with the recent studies on graphene adsorption mechanisms, where the formation of a solvent monolayer, as discussed in [21], plays a crucial role in modulating the interaction between solutes and graphene surfaces. This behavior is particularly important for understanding how graphene oxide influences molecular interactions in complex solvent environments.

**Figure 13:**
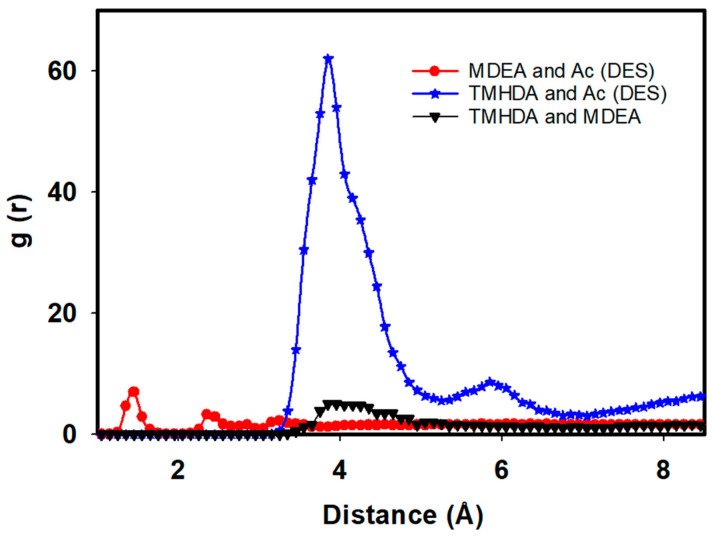
RDF between DES components supported by graphene oxide during desulfurization of natural gas.

Figure 13 illustrates the RDF between DES components supported by graphene oxide during the desulfurization process. The data indicate that the DES structure remains stable throughout the simulation, which is crucial for the continuous and effective removal of hydrogen sulfide.

**Figure 14:**
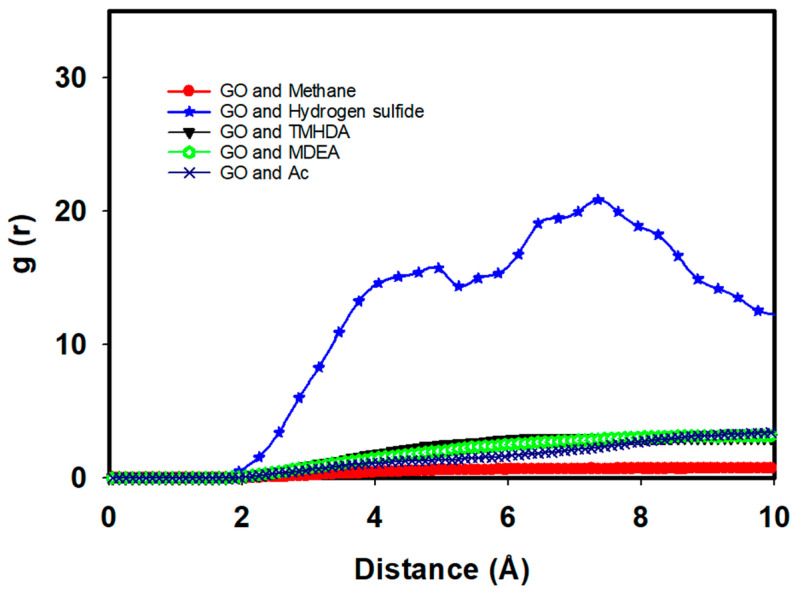
RDF between graphene oxide (GO) and other components during desulfurization of natural gas using DES.

To further elucidate the interactions between GO and the components involved in the desulfurization of natural gas using DES, we analyzed the radial distribution function (RDF) (Figure 14) between GO and various gas species, including methane, hydrogen sulfide, TMHDA, MDEA, and Ac. Figure 14 presents the g(r) curves for these interactions, showing distinct peaks that highlight the spatial distribution of the components around the GO nanosheet. Notably, the RDF curve for hydrogen sulfide demonstrates a prominent peak at approximately 4–6 Å, indicating stronger affinity between GO and H₂S compared to other components. This suggests that GO plays a significant role in capturing hydrogen sulfide, while the interactions with methane and other species exhibit relatively lower g(r) values, supporting weaker adsorption on GO. The differences in the peaks of the g(r) curves reinforce the selective affinity of GO towards sulfur-containing species, which is crucial for the efficient desulfurization process. These nanosheet–component interactions complement the bulk–bulk interactions of the system, as previously discussed, and provide insights into the specific role of GO in enhancing the overall performance of the DES in the natural gas desulfurization process.

The stability of the DES is likely a result of the strong interactions between its components, as evidenced by the high peak values in the RDFs and the significant adsorption energies reported in Table 6.

Table 6 provides a quantitative assessment of the interactions between hydrogen sulfide and various DES components. The adsorption energy between hydrogen sulfide and acetate is particularly high at −362.43 kJ/mol, suggesting a very strong interaction. This strong interaction is likely due to the formation of hydrogen bonds as acetate is a highly polar molecule capable of donating and accepting hydrogen bonds. The interactions between hydrogen sulfide and TMHDA (−28.63 kJ/mol) and MDEA (−24.47 kJ/mol) are also significant, albeit weaker than with acetate, indicating that these components also contribute to the overall desulfurization process.

Table 7 further corroborates these findings by listing the number of hydrogen bonds between hydrogen sulfide and DES components. The highest number of hydrogen bonds is observed between acetate and TMHDA (715), followed by MDEA and TMHDA (823). These hydrogen bonds are critical for the stability and effectiveness of the DES in capturing hydrogen sulfide. The high number of hydrogen bonds between MDEA and TMHDA also suggests that these components work synergistically to stabilize the DES structure and enhance its desulfurization capability.

The MD simulation results provide compelling evidence of the effectiveness of the task-specific DES system supported by graphene oxide in the desulfurization of natural gas. The strong interactions between the DES components and hydrogen sulfide, coupled with the stability of the DES structure, suggest that this system is highly effective in selectively removing hydrogen sulfide from methane. The presence of graphene oxide further enhances the structural integrity and overall performance of the DES, making it a promising approach for natural gas purification. This work can also be further studied by creating digital twin models for the natural gas desulfurization process.

## 3. Methodology

### 3.1. Theoretical Models

**Figure 15:**
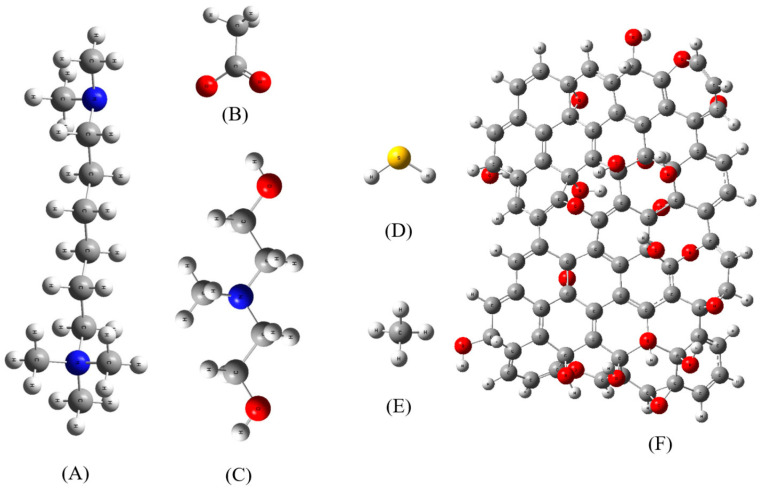
(**A**) [C1-TMHDA], (**B**) Ac, (**C**) MDEA, (**D**) hydrogen sulfide, (**E**) methane, and (**F**) graphene oxide structures for our calculations. Color legend: white represents hydrogen; gray indicates carbon; blue denotes nitrogen; yellow denotes sulfur; and red signifies oxygen.

[C1-TMHDA]Ac-MDEA (1:1) was chosen as the theoretical representation of task-specific DESs supported by graphene oxide, while methane with hydrogen sulfide was selected as a model representative of natural gas for our DFT calculations and traditional all-atom MD simulations. The specific structure of the task-specific DES supported by graphene oxide, as depicted in Figure 15, served as the computational model for natural gas desulfurization using DFT calculations and classical all-atom MD simulations.

Following model selection, we chose to conduct DFT calculations and classical all-atom MD simulations involving a single task-specific DES supported by graphene oxide interacting with methane in the presence of hydrogen sulfide as an impurity.

### 3.2. DFT Calculations

For our DFT computations, we used typical structures of methane, hydrogen sulfide, and task-specific DES in the absence of graphene oxide, as shown in Figure 15. It is significant to remember that in the presence of methane, the binding structures of hydrogen sulfide with the task-specific DES are not electrically neutral. Our study investigated the covalent and noncovalent interactions between task-specific DES components and hydrogen sulfide in the presence of methane. The B3LYP/6-311+G(d,p) level of theory was utilized to fully optimize the intermolecular interaction of the task-specific DES and its application in natural gas desulfurization in the gas phase [22,23].

In order to understand the intermolecular interaction of task-specific DESs and their applications in the desulfurization of natural gas, quantum chemical features such as optimal structures and molecular orbitals were acquired after optimization in the gas phase. All stationary points were also verified as genuine minima on their corresponding potential energy surfaces through an analytical computation of the second energy derivatives. To compute electronic ground state geometries, DFT calculations were performed using Gaussian16 with GausView (v6.0) [24].

### 3.3. Classical All-Atom MD Simulations

Figure 15 shows the segment model representing the task-specific DES as [C1-TMHDA]Ac-MDEA and graphene oxide. A model depicting natural gas was built using methane with hydrogen sulfide as an impurity. Lennard-Jones (LJ) parameters were taken from the standard GROMOS force field, while force field parameters (dihedrals, angles, bonds, and partial charges) and coordinates for various functional groups (Figure 15) were acquired from the ATB database [25]. Then, representative sections of task-specific DES systems supported by graphene oxide were created for MD simulations, both with and without natural gas components such as hydrogen sulfide and methane. The potential energy of the intended system, including bound and non-bonded contributions, was computed using the GROMOS force field [26].

After building the first simulation box for each system, the configuration was optimized at 298 K and 1 bar pressure for 0.1 ns through the use of the steepest descent approach for energy reduction. Following that, NPT and NVT equilibrations were carried out for 1 ns each at 298 K and 1 bar of pressure. This produced an equilibrated box size of 10 × 10 × 10 nm^3^. Molecular Dynamics simulations were run under the NVT ensemble for 10 ns at 298 K and 1 bar after equilibrium was reached. All bonds were subjected to the LINCS constraint algorithm during the simulation, with a 1.0 nm cut-off for coulombic short-range interactions and LJ. Using fourth-order interpolation and 0.16 nm grid spacing, Particle Mesh Ewald summation was used to calculate long-range interactions. Temperature was maintained using the V-rescale technique, while system pressure was maintained using Berendsen pressure coupling. All directions were subjected to periodic boundary conditions [27,28,29,30].

All-atom MD simulations were performed using GROMACS version 2019.6 software; the simulated box was visualized using Visualization Molecular Dynamics (VMD), and the free energy surface was plotted using a Sigma plot, respectively [31]. The density of the simulation box was kept at roughly 1 g/cm^3^ at 298.15 K, 1 bar for all simulations, indicating a good agreement between the experimental and computed data (standard deviation < 2%) and supporting the force field selection. The choice of force field parameters is important in MD simulation.

## 4. Conclusions

In conclusion, our comprehensive study on the desulfurization of natural gas using a task-specific Deep Eutectic Solvent (DES) composed of TMHDAAc and MDEA, supported by graphene oxide, offers valuable atomistic insights into the underlying mechanisms of hydrogen sulfide removal from methane. Through a combination of Density Functional Theory (DFT) calculations and classical all-atom Molecular Dynamics (MD) simulations, we elucidated the complex interactions, structural stability, and molecular dynamics that contribute to the efficiency of this DES system in the desulfurization process.

The DFT calculations, particularly the optimized structural and HOMO-LUMO orbital analyses, highlight the distinct spatial arrangements and interactions between hydrogen sulfide, methane, and the DES components. These findings emphasize the ability of the task-specific DES to facilitate the separation of hydrogen sulfide from methane effectively, indicating strong selective interactions and chemical affinities. The insights gained from the DFT-based orbital analyses provide a clear understanding of the electron density distributions, which are critical for understanding how the DES components interact with hydrogen sulfide on a molecular level.

In parallel, the traditional all-atom MD simulations offer detailed insights into the dynamic behavior and interactions of the DES in a natural gas environment. The radial distribution function (RDF) analyses reveal the specific distances and spatial correlations between hydrogen sulfide and the DES components, while the interaction energy calculations quantify the strength and specificity of these interactions. Importantly, the presence of graphene oxide as a supporting material enhances the structural stability of the DES, as confirmed by the MD simulation results, which show that the DES remains robust even after mixing with methane. This ensures the suitability of the DES for prolonged desulfurization processes under industrial conditions.

Furthermore, the ability of the DES to maintain its structural integrity and continue performing effectively over extended periods underscores its potential for practical applications in sustainable natural gas purification. The synergy between TMHDAAc, MDEA, and graphene oxide provides a robust framework for the design of advanced desulfurization systems capable of meeting industrial requirements for efficiency, durability, and environmental sustainability.

These results collectively demonstrate that the task-specific DES supported by graphene oxide is not only highly effective at removing hydrogen sulfide from methane but also stable enough for long-term use. The atomistic insights derived from this study offer a strong foundation for the future optimization of DES systems, potentially leading to more efficient, scalable, and environmentally friendly solutions for natural gas purification. This work provides critical guidance for the development of next-generation DES systems tailored for specific gas separation applications, contributing to the advancement of sustainable energy technologies. Moreover, the development of machine learning and artificial intelligence could help us further advance digital twins for the desulfurization of the natural gas process.

## Figures and Tables

**Table 1:** The energy for the DES and its components and the bond lengths between the interacting fragments of the DES.

	TMHDAAc	MDEA	Task-Specific DES	Excess Energy
Total Energy (eV)	−21,050.72	−10,983.88	−32,034.43	0.17
	O atom of Ac and H atom of TMHDA	O atom of MDEA and H atom of TMHDA		O atom of Ac and H atom of MDEA
Bond Length (Å)	2.17	2.62		2.44

**Table 2:** Intermolecular interactions between DES components.

Interactions	Energy (SD) (kJ/mol)
TMHDA and AC (Pure)	−10,843.2 (±380.51)
TMHDA and Ac (DES)	−9895.71 (±325.39)
TMHDA and MDEA (DES)	−1828.51 (±73.02)
MDEA and AC (DES)	−10,783.8 (±415.43)

**Table 3:** Numbers of hydrogen bonds between DES components.

H Bond	Number of H Bonds (SD)
TMHDA and AC (Pure)	690 (±26)
TMHDA and Ac (DES)	612 (±18)
TMHDA and MDEA (DES)	63 (±3)
MDEA and AC (DES)	383 (±14)

**Table 4:** Adsorption energy between hydrogen sulfide and DES components.

Interactions	Energy (SD) (kJ/mol)
H_2_S and Methane (Pure)	−17.23 (±0.72)
H_2_S and Methane (DES)	−0.82 (±0.04)
H_2_S and Ac	−135.24 (±4.83)
H_2_S TMHDA	−11.96 (±0.46)
H_2_S MDEA	−38.21 (±1.36)
Ac and TMHDA	−10,403.53 (±376.12)
Ac and MDEA	−6858.04 (±249.03)
MDEA and TMHDA	−1367.23 (±48.89)

**Table 5:** Number of hydrogen bonds between hydrogen sulfide and DES components.

H Bond	Number of H Bonds (SD)
Ac and TMHDA	670 (±24)
Ac and MDEA	260 (±8)
MDEA and TMHDA	590 (±32)

**Table 6:** Adsorption energy between hydrogen sulfide and DES components supported by graphene oxide.

Interactions	Energy (kJ/mol)
H_2_S and Methane (Pure)	−17.23 (±0.62)
H_2_S and Methane (DES)	−0.56 (±0.03)
H_2_S and Ac	−362.43 (±12.86)
H_2_S TMHDA	−28.63 (±1.02)
H_2_S MDEA	−24.47 (±0.87)
Ac and TMHDA	−8091.71 (±293.21)
Ac and MDEA	−6305.90 (±230.71)
MDEA and TMHDA	−2743.35 (±98.02)

**Table 7:** Number of hydrogen bonds between hydrogen sulfide and DES components supported by graphene oxide.

H Bond	Number of H Bonds
Ac and TMHDA	715 (±28)
Ac and MDEA	251 (±8)
MDEA and TMHDA	823 (±30)

## Data Availability

The datasets generated during and/or analyzed during the current study are available from the corresponding author upon reasonable request.
